# Association between Turbot (*Scophthalmus maximus*) Fish Phenotype and the Post-Larval Bacteriome

**DOI:** 10.3390/microorganisms12102014

**Published:** 2024-10-04

**Authors:** Antonio Louvado, Davide A. M. Silva, Vanessa Oliveira, Carolina Castro, Daniel F. R. Cleary, Newton C. M. Gomes

**Affiliations:** 1 CESAM & Department of Biology, University of Aveiro, 3810-193 Aveiro, Portugal; antonio.louvado@ua.pt (A.L.); dams@ua.pt (D.A.M.S.); v.oliveira@ua.pt (V.O.); cleary@ua.pt (D.F.R.C.); 2 Flatlantic S.A., 3070-732 Praia de Mira, Portugal; ccastro@flatlantic.pt

**Keywords:** larviculture, microbiome, skeletal abnormalities, malpigmentation, Cytophagales flatfish

## Abstract

Over the past decade, an increasing number of studies have emphasized the importance of the host microbiome in influencing organismal health and development. Aligned with this understanding, our study aimed to investigate the potential association between the turbot (*Scophthalmus maximus*) phenotypic traits and the post-larval bacteriome. Turbot post-larvae were sampled from twenty randomly selected production cycles thirty days after hatching (DAH) across multiple post-larval production batches over a three-month period (April to June). Fish were selectively sampled based on five phenotypic traits, namely, normal, large, small, malformed, and depigmented. Our results showed that small-sized post-larvae had significantly higher bacterial phylogenetic diversity in their bacterial communities than all other phenotypes. A more in-depth compositional analysis also revealed specific associations between certain bacterial taxa and fish phenotypes. For example, the genera *Aliivibrio* and *Sulfitobacter* were enriched in small-sized post-larvae, while the family Micrococcaceae were predominantly found in larger post-larvae. Furthermore, genus *Exiguobacterium* was linked to depigmented larvae, and genus *Pantoea* was more prevalent in normal post-larvae. These observations underscore the importance of further research to understand the roles of these bacterial taxa in larval growth and phenotypic differentiation. Such insights could contribute to developing microbiome modulation strategies, which may enhance turbot post-larval health and quality and improve larviculture production.

## 1. Introduction

Over the past two decades, extensive research has focused on optimizing diet and rearing conditions to enhance larval health and minimize the incidence of undesirable phenotypic traits in larviculture systems [1,2,3,4]. Currently, larviculture production relies on optimized protocols to produce a high yield of high-quality fingerlings for the grow-out phase [5]. Despite improvements in nutrition, feeding regimes, and rearing conditions, a large percentage of post-larvae remain underdeveloped (small) or have abnormal features such as skeletal malformation or an abnormal skin pigmentation [6,7]. Fish produced in aquaculture systems tend to exhibit a higher incidence of malformations and depigmentation compared to their wild counterparts [8,9]. These abnormal features are often attributed to genetics, an improper dietary regime, or rearing conditions [5]. Fish exhibiting malformations may not survive or grow poorly in the production system, whereas the presence of abnormal pigmentation can downgrade the image of the product to consumers, with both traits ultimately resulting in economic losses [10,11].

Fish microbial communities play a pivotal role in nutrient metabolism, immunomodulation, protection against pathogens (e.g., through competition and antagonism), and potentially exert host transcriptional effects (e.g., cell differentiation or activation of the immune system) [12,13,14]. Motivated by the importance of improving our understanding of host–microbe interactions in organismal health, there is currently a growing body of research aimed at elucidating these interactions and their impact on fish development and health [12,13,14]. For example, previous studies have proposed that the feed supplementation with probiotics and chemical microbiome modulators (e.g., prebiotics) can promote fish growth and immunity and mitigate skeletal malformations [9,15,16,17,18,19,20,21].

At early life-stages, the host microbiome is crucial to ensuring a balanced immune response by training the host’s immune system to recognize potential pathogenic microbes while tolerating beneficial ones [22]. Meanwhile, the initial gut microbiome, by ensuring a more thorough digestion of complex macromolecules and the synthesis of vitamins, amino acids, organic acids, and growth metabolites, aides nutritional uptake and overall growth [23]. During early development, the microbiome also influences organ and skeletal development. Previous studies have shown that microbiota disruption, in early life, can alter host organ and tissue development [24]. For example, germ-free hosts had a reduced rate of epithelial proliferation and increased intestinal transit rate, and reduced harvest of energy from feed than non-germ-free hosts [25,26]. Interestingly, Lamari et al. (2013) highlighted the potential of different probiotics on fish bone mineralization, including both positive and negative effects on fish development [16].

In contrast to skeletal malformation, few studies have focus on the potential role of microbes on fish pigmentation [27,28]. Fish malpigmentation is an abnormal pattern or intensity of skin pigmentation that can occur in both wild and cultured fish populations. While genetic factors can predispose certain fish species or individuals to pigmentary disorders [29], environmental factors, particularly diet and rearing conditions, are known to play an important role in their manifestation and severity [28]. Notably, pigmentation disorders are prevalent in flatfish during intensive larviculture, and several strategies have been developed to reduce the occurrence of this disorder. These strategies include dietary optimization [30,31] and manipulation of rearing conditions such as light intensity, temperature, and stocking density [2,32,33]. However, despite previous studies suggesting a potential link between fish pigmentation and alterations to skin and gut structure, as well as changes in the gut microbiome [28], the association between the fish microbiome and pigmentation remains largely unexplored.

A better understanding of fish–microbe interactions can lead to significant advancements in aquaculture production promoting healthier and more resilient fish with desirable traits. Aquaculture productivity, particularly in the finfish sector, is chronically hampered by undesirable traits. Estimates indicate that malformations alone can affect 7 to 20% of a cohort [34] with economic losses ranging from EUR 20 to 100 million across the whole of the Mediterranean aquaculture sector [35]. In this study, we aimed to investigate the potential associations between the bacteriome of post-larval turbot (*Scophthalmus maximus*) and the phenotypic traits observed across different culture batches in intensive larviculture production. To achieve this goal, we analyzed the bacterial communities of 30-day-old turbot post-larvae (whole fish) with five distinct phenotypes (normal, large, small, malformed, and depigmented), collected from twenty randomly selected production cycles across multiple post-larvae production batches in an intensive larviculture system.

## 2. Material and Methods

### 2.1. Sampling and DNA Extraction

Fish were sampled between the 22nd of April and the 3rd of July 2022 in an intensive Portuguese larviculture facility for the production of turbot (*S. maximus*) larvae. This larviculture operates a flow-through system, where natural seawater is filtered and UV-sterilized. Fish larvae and post-larvae rearing were conducted as part of routine animal husbandry practices in commercial farming operations as previously described [36]. Briefly, from 3 to 15 days after hatching (DAH), larvae were fed with rotifers (*Brachionus plicatilis*) along with a mature culture of *Tetraselmis* sp. using the green water technique. Between 11 and 15 DAH, rotifers were gradually replaced with brine shrimp (*Artemia* sp.). From 16 to 22 DAH, the post-larvae were fed exclusively with brine shrimp. Finally, from 22 to 30 DAH, brine shrimp were gradually replaced with dry feed. Additional information on the production parameters used in the various larviculture production cycles is available in Supplementary File S1.

Eighty post-larvae (30 days after hatching, DAH) were collected from twenty larviculture production cycles across five production batches. Each batch comprised production cycles reared simultaneously under identical conditions. From each production cycle, five fish were randomly selected, representing the following phenotypic categories:

(1) Normal (Nor): Fish of average size with normal pigmentation and no malformations.
(2) Large (Lrg): Larger than average fish with normal pigmentation and no malformations.
(3) Small (Sml): Smaller than average fish with normal pigmentation and no malformations.
(4) Malformed (Maf): Fish of average size with normal pigmentation but exhibiting skeletal malformation.
(5) Depigmented (Dep): Fish of average size with abnormal pigmentation and no malformations.

Skeletal malformations and skin pigmentation were assessed using visual observation. Fish size was assessed by measuring the tail to mouth length. First, thirty individuals from each production cycle were measured to determine a Gaussian distribution model of fish length in the cohort. From this model, thresholds for small and large fish were determined for each production cycle at the 5th and 95th percentile values, respectively. Overall, the length of fish from the Sml category ranged from 14 to 16 mm; DeP, Nor, and Maf ranged from 16 to 26 mm; and Lrg ranged from 24 to 31 mm (Supplementary File S1). Photographs of example specimens from each category are presented in Supplementary Figure S1 in the Supplementary File S3. Samples were collected as part of routine husbandry practices in commercial farming operations, with no experimental procedures conducted on live fish. The sampling followed animal welfare protocols in accordance with Annex IV of the European Directive 2010/63/EU on the protection of animals used for scientific purposes. Turbot post-larvae were sampled and euthanized using an overdose of fish anesthetic (clove oil; Sigma-Aldrich, St. Louis, MO, USA). After this procedure, each fish was rinsed with sterile artificial seawater and stored in ethanol at −20 °C until DNA extraction. DNA extraction was performed on whole post-larvae using the FastDNA^TM^ Spin kit (MP Biomedicals, Irvine, CA, USA) following the manufacturer’s instructions. A blank negative control with no sample was included in the DNA extraction process for further analysis.

### 2.2. High-Throughput Sequencing Data Acquisition

The hypervariable region V3/V4 of the 16S rRNA gene was amplified by PCR using the primers 314F (CCTACGGGNGGCWGCAG) and 785R (GACTACHVGGGTATCTAATCC). Library preparation and sequencing were performed by Molecular Research LP (www.mrdnalab.com; Shallowater, TX, USA) on a MiSeq sequencing platform following standard Illumina procedures (Illumina, San Diego, CA, USA). Algorithms in QIIME2 (version 2020.8) were used to transform the amplicon libraries to an amplicon sequence variant (ASV) count table [37]. Demultiplexing was performed using the “demux” algorithm in QIIME2. The dada2 algorithm from the DADA2 plugin [38] in QIIME2 was used to filter low-quality reads, merge forward and reverse reads, remove chimeras, and generate ASVs. In DADA2, forward and reverse sequences were truncated at 220 and 240 bp, respectively. Taxonomy was assigned to ASVs using the “feature-classifier” algorithm in QIIME2 with a scikit-learn Naïve Bayes classifier based on the SILVA database of the 16S reference sequences at 99% similarity (version 138, released December 2019). The classifier was previously trained using a “feature-classifier” algorithm in QIIME2 (version 2020.8) with reference sequences trimmed and truncated at the 314F and 785R region. To simplify interpretation, a unique number was assigned to each ASV. Non-bacterial, mitochondrial, and chloroplast sequences were removed. ASVs that occurred in the negative controls, but which did not appear to be the result of “index hopping”, were removed [39].

### 2.3. Data Analysis and Statistics

A table containing the ASV counts per sample (Supplementary File S2) was imported into R and used to compare the diversity, composition, and predicted function of bacterial communities. The Shannon H’ diversity index was calculated using the diversity() function in the vegan package [40], evenness (Pielou’s J) was calculated by dividing Shannon H’ by the logarithm of the number of ASVs, and Fisher’s alpha was calculated using the fisher.alpha() function in vegan. Faith’s phylogenetic diversity index (PDI) was calculated using the pd() function from the picante package [41] with the input consisting of the ASV count table and a rooted phylogenetic tree in R. The phylogenetic tree was created using the FastTree algorithm [42] from the phylogeny plugin in QIIME2 with a MASFFT alignment [43] of valid sequences. We tested for deviations from normality using the Shapiro–Wilk test and found that the diversity parameters deviated significantly (*p* < 0.05). We, therefore, used the Kruskal–Wallis rank sum test using the kruskal.test() function from the stats package to test for significant differences in the diversity indices among categories. If the Kruskal–Wallis test yielded a significant result, we subsequently applied post hoc analysis with Dunn’s nonparametric test of multiple comparisons [44] with *p*-values adjusted using the Benjamini–Hochberg procedure (false discovery rate). Dunn’s test was calculated using the DunnTest() function from the FSA package in R [45].

Biplot ordinations of ASV composition were produced using phyloseq with the phyloseq() function in the phyloseq package [46]. The ordinate() function in from the phyloseq package was subsequently used with the phyloseq()-generated object as input. The method argument was set to “PCoA”, and the distance argument was set to “bray”. A biplot was then produced using the plot_ordination() function with the type argument set to “biplot”. We tested for significant differences in ASV composition among categories using the adonis() function from the vegan package [40]. Variation in the relative abundances of selected bacterial higher taxa among categories (Sml, Nor, DeP, Maf, and Lrg) were tested for significance using the ANOVA f-test applied to generalized linear models (GLM). The anova() function in R was applied to a GLM model generated with the glm() function in R for each selected taxon. Since a number of these variables included an excess of zero counts in the samples, we set the family argument to “tweedie” using the tweedie() function in the statmod package in R with var.power = 1.5 and link.power = 0 (a compound Poisson–gamma distribution). Post hoc analysis of taxa that varied significantly (*p*-value < 0.05) among categories was assessed using estimated marginal means models created with the emmeans() function from the emmeans package in R with the p adjustment set to the false discovery rate (i.e., p.adjust = “fdr”) [47]. Pairwise comparisons were performed by calculating the estimated marginal means of GLM models, which related these effects to the specific categories and their combinations using pairs() function from the emmeans package with *p*-value adjustment set to the false discovery rate (i.e., p.adjust = “fdr”). The BLAST search tool (http://www.ncbi.nlm.nih.gov/, accessed on 1 March 2024) was used to compare representative sequences of the 50 most abundant ASVs of each organism with sequences in NCBI’s 16S ribosomal RNA (Bacteria and Archaea type strains) database using standard parameter settings. Sequences that exhibited the highest levels of similarity to sequences of our ASVs were considered closely related organisms.

## 3. Results and Discussion

**Figure 1:**
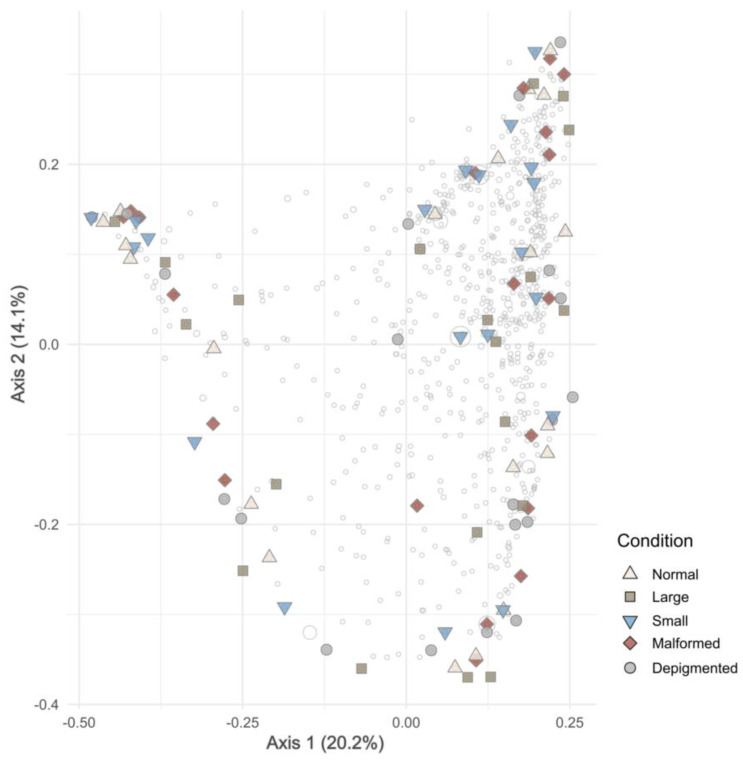
Ordination showing the first two axes of the principal coordinates analysis (PCoA) of ASV composition. Percentage represents the proportion of variance of each axis as calculated by its eigenvalue. Symbols are color-coded and represent samples from the different categories. Malformed: normal size fish with normal pigmentation and at least one detectable skeletal malformation; Small: small size fish with normal pigmentation with no skeletal malformation; Depigmented: normal size fish with irregular pigmentation and no skeletal malformation; Large: large size fish with normal pigmentation and no skeletal malformation; and Normal: normal size fish with normal pigmentation and no skeletal malformation. Grey symbols represent weighted average scores for ASVs. The symbol size is proportional to abundance (number of sequences reads).

**Figure 2:**
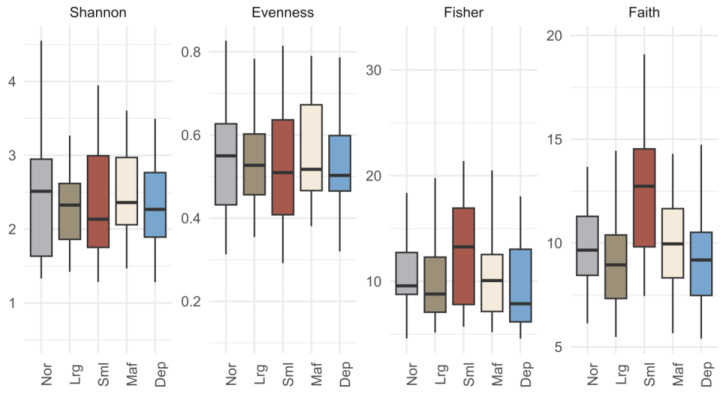
Boxplots of diversity indices Pielou’s J (evenness), Shannon’s H’, Fisher’s alpha, and Faith’s phylogenetic diversity for each category. Maf: normal size fish with normal pigmentation and at least one detectable skeletal malformation; Sml: small size fish with normal pigmentation with no skeletal malformation; Dep: normal size fish with irregular pigmentation and no skeletal malformation; Lrg: large size fish with normal pigmentation and no skeletal malformation; and Nor: normal size fish with normal pigmentation and no skeletal malformation.

After quality control and removal of non-bacterial sequences, the dataset consisted of more than 3 million sequences grouped into 8513 ASVs. The PCoA analysis of ASV composition did not reveal any distinct clustering of samples based on fish categories (Figure 1). This lack of separation was further corroborated by a PERMANOVA analysis (F_4,99_ = 0.876, *p* = 1). The diversity parameters Shannon’s ‘H, Peilou’s J (evenness) and Fisher’s α did not differ significantly (Kruskal–Wallis: *p* > 0.05) among categories (Nor, Lrg, Sml, Maf, and Dep) (Figure 2 and Table S1 in Supplementary File S3). However, there was a significant difference in Faith’s PDI among categories (Table S1 in Supplementary File S3). Post-hoc multiple comparisons using Dunn’s Z test revealed that bacterial communities of fish from the Sml category had a significantly higher phylogenetic diversity than fish from all other categories (Dunn Test: *p* < 0.05; Table S2 in Supplementary File S3). These results are intriguing and indicate that Sml fish comprised bacterial populations with greater “evolutionary distance” among themselves compared to the communities associated with other fish phenotypes. Probably, particular biological features of smaller post-larvae, for example incomplete development or other metabolic issues, may have influenced fish bacterial diversity.

**Figure 3:**
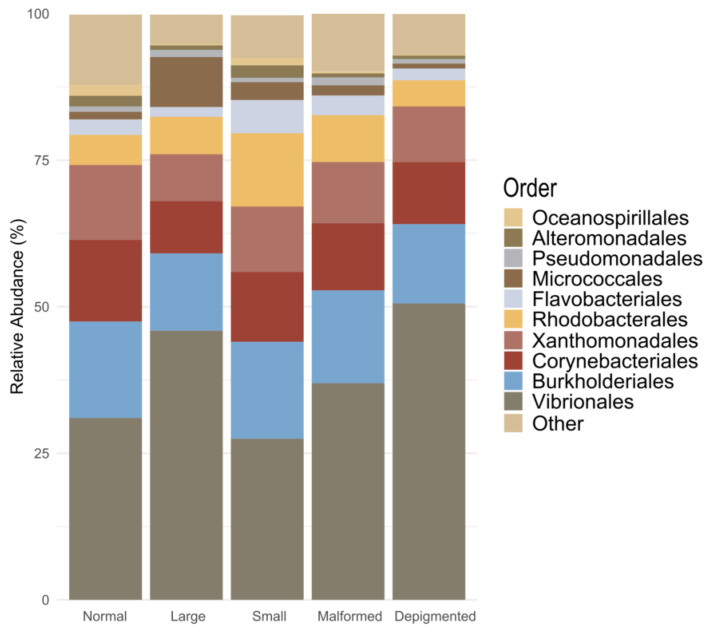
Stacked barplot showing the mean relative abundances of the ten most abundant orders in the dataset. Malformed: normal size fish with normal pigmentation and at least one detectable skeletal malformation; Small: small size fish with normal pigmentation with no skeletal malformation; Depigmented: normal size fish with irregular pigmentation and no skeletal malformation; Large: large size fish with normal pigmentation and no skeletal malformation; and Normal: normal size fish with normal pigmentation and no skeletal malformation.

**Figure 4:**
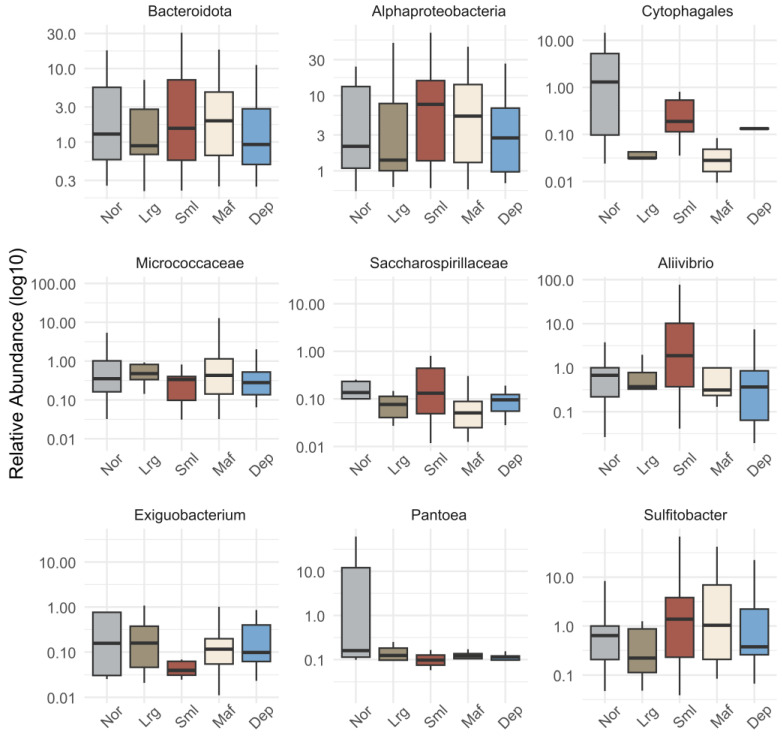
Boxplots of the relative abundances of significantly enriched taxa (EMMEANS *p* < 0.005; Supplementary File S4). Only taxa that significantly differed in relative abundance in comparison to the Nor category were considered. Supertaxon that were considered redundant were excluded. Maf: normal size fish with normal pigmentation and at least one detectable skeletal malformation; Sml: small size fish with normal pigmentation with no skeletal malformation; Dep: normal size fish with irregular pigmentation and no skeletal malformation; Lrg: large size fish with normal pigmentation and no skeletal malformation; and Nor: normal size fish with normal pigmentation and no skeletal malformation. Y-axis is a logarithmic scale.

Previous studies have shown that fish bacterial communities can be shaped by a range of host biological factors (e.g., genetics, age, metabolic demands, and immune system) [48,49]. However, the host phenotypes may be influenced by the combined effects of host and microbial genes, referred to as metagenomic effects, rather than solely by dominant genes from either the host or microbiome [50]. For example, studies in mice have successfully identified alterations at specific loci in the mouse genome associated with specific microbial taxa in the gut [51]. Overall, the post-larvae bacterial communities were dominated by ASVs affiliated with the proteobacterial orders Vibrionales, Burkholderiales, Xanthomonadales, Rhodobacterales, and Corynebacteriales (Figure 3). A detailed compositional analysis of the most abundant taxa (mean relative abundance > 1% in any category) revealed significant variation in the relative abundances of bacterial groups among categories (GLM-ANOVA: *p* < 0.05; Table S3 in Supplementary File S3). We subsequently ran pairwise tests for each taxon, which yielded significant differences among categories with a focus on differences between the Nor and other categories (Supplementary File S4). In Figure 4, we show the relative abundances of higher taxa that differed significantly among categories (Table S3 in Supplementary File S3; ANOVA: *p* < 0.05) and differed significantly with respect to the Nor category in pairwise comparisons (Supplementary File S4; EMMEANS *p* < 0.05; supertaxon considered as redundant were not included in Figure 4). Our analysis revealed a significant difference in the relative abundance of the phylum Bacteroidota between the Nor and Lrg categories (EMMEANS: *p* < 0.05; Supplementary File S4). Specifically, Nor samples had a higher abundance of Bacteroidota ASVs compared to Lrg samples (Figure 4). Bacteroidota is a versatile phylum, prevalent across various aquatic and terrestrial biotopes with an important role in the turnover of organic matter [52]. The observed differential abundance of Bacteroidota between the Nor and Lrg categories was primarily related to the order Cytophagales. The relative abundance of this order differed significantly among categories and in relation to the Nor category. Subsequent analysis revealed that Cytophagales was predominantly represented by ASV-45, which was 97.16% similar to the type strain *Bernardetia litoralis* DSM 6794 (formerly *Flexibacter litoralis* DSM 6794; Table S4 in Supplementary File S3). This strain was originally isolated from aquarium seawater [53]. This bacterium, along with other species belonging to the Bernardetiaceae family, is frequently detected in various marine environments, including bivalve hatchery systems [54].

Our results also showed significant enrichment of ASVs related to Alphaproteobacteria and *Aliivibrio* in the Sml compared to the Nor category (EMMEANS: *p* < 0.05; Supplementary File S4, Figure 4). The observed variation in alphaproteobacterial sequences was partly attributed to the elevated abundance of ASV-57, identified as belonging to the genus *Sulfitobacter* (Table S4 in Supplementary File S3). This ASV had 100% sequence similarity to *Sulfitobacter* sp. SJ1724 (KF477333), a strain isolated from seawater near coral reefs in the Norwegian Sea (Table S4 in Supplementary File S3) [55]. In the post hoc analysis, the abundance of the genus *Sulfitobacter* also differed significantly in the Nor–Sml comparison (Supplementary File S4), with a tendency of lower relative abundances in the Lrg category. Members of this genus are known as one of the primary sulfite-oxidizing marine bacteria, found ubiquitously across various marine habitats colonizing algae, animal hosts, and environmental biotopes [56]. *Sulfitobacter* has previously been suggested to be a potentially beneficial marine bacterium due to its antagonistic activity against *Vibrio anguillarum* [57]. In our study, the enrichment of *Aliivibrio* in Sml post-larvae was linked to ASVs 8867 and 2440. Both ASVs had high sequence similarities to *Aliivibrio fischeri* strains previously isolated from marine animal hosts, such as the *Euprymna scolopes* squid (Table S4 in Supplementary File S3) [58]. The genus *Aliivibrio* is a distinct group within the family Vibrionaceae created by Urbanczyk et al. (2007) after reclassification of the former *Vibrio fischeri* clade [59]. *Aliivibrio fischeri* is known to be bioluminescent and to establish symbiotic relationships with animal hosts [60,61]. Although *Aliivibrio* strains are commonly associated with non-pathogenic symbiotic relationships, some studies have linked members of this genus, including *A. fischeri*, to diseases in farmed fish [62].

Post-larvae with depigmentation exhibited a slightly but significantly higher relative abundance of *Exiguobacterium* compared to those in the Nor category (EMMEANS *p* < 0.05, Figure 4, Supplementary File S4). *Exiguobacterium* is a versatile bacterial genus of pigmented, Gram-positive bacteria that have been isolated from a multitude of terrestrial and marine environments [63,64], including aquaculture rearing water, live feed cultures, farmed gilt-head seabream tissues, and aquaculture shrimp ponds [65,66,67,68]. In our dataset, ASV-4250 was identified as the most abundant ASV classified to the genus *Exiguobacterium* and was closely related to *Exiguobacterium qingdaonense* strain S82, isolated from *Sargassum* in the Gulf of Tonkin (Table S4 in Supplementary File S3) [64]. *Exiguobacterium strains* are usually associated with healthy fish and, owing to their antagonistic potential against fish pathogens [69,70], certain strains have been suggested as potential probiotics for aquaculture settings [71,72]. However, no previous study has investigated their potential influence on fish pigmentation. Currently there is a significant knowledge gap regarding the interplay between microbial communities and fish pigmentation. Pinto et al., 2019 demonstrated that pseudo-albinism, in addition to other pigmentation abnormalities, was associated with alterations in the structure of the skin and gut of sole (*Solea senegalensis*) and a modification in the gut microbiome [28]. Interestingly, they observed significantly lower abundances of *Mycoplasma* and *Vibrio* in pseudo-albino sole compared to pigmented sole. However, contrary to their findings, our study indicates no significant shifts in the abundances of these genera among post-larvae from the Dep category.

Our analysis further identified that the Lrg category exhibited significantly higher abundances of Micrococcaceae ASVs than the Nor category (EMMEANS *p* < 0.05; Supplementary File S4, Figure 4). The Micrococcaceae differential abundance in Lrg samples is mainly attributed to a single ASV with 100% similarity to *Pseudarthrobacter phenanthrenivorans* and several other *Arthrobacter* strains in the NCBI database (Table S4 in Supplementary File S3). The current understanding of the impact of members of the Micrococcaceae family on fish aquaculture production is ambiguous, with different studies associating this family to both healthy and unhealthy fish states [14,73,74]. Nevertheless, previous studies have suggested that members of this family can produce antimicrobial compounds and serve as fish probiotics [73,75,76]. Strains belonging to the *Pseudarthrobacter* and *Arthrobacter* genera have frequently been detected in aquaculture, yet their functional role is speculative. Novel strains of *Arthrobacter* have been isolated from skin ulcers in salmonoids in their freshwater life cycle stage, yet proof of pathogenicity was not presented [77]. In shrimp aquaculture, two *Arthrobacter* strains have been isolated and found to possess potential *Vibrio* antagonism [78,79]. Tested alongside an effective antibiotic, *Arthrobacter* XE-7 was found to have comparable attenuation rates of pathogenic *Vibrio* strains in shrimp (*Penaeus chinensis*) post-larvae [79].

Our results also showed that the Nor category hosted higher relative abundances of Saccharospiraceae (basonym Oceanospirillaceae), and *Pantoea* spp. (Figure 4) compared to all other categories, with the exception of the Small category for Saccharospiraceae. The differential abundance of the family Saccharospiraceae (basonym Oceanospirillaceae) was mainly due to ASV-68 (Table S4 in Supplementary File S3), an ASV classified as an uncultured marine organism and most similar (97.66%) to a *Spongiispira norvegica* symbiont previously detected in the boreal sponge *Isops phlegraei* [80]. Strains belonging to the Saccharospiraceae (basonym Oceanospirillaceae) family are not typically detected in aquaculture settings. Microbes classified to this family have been repeatedly detected in marine environmental settings, mainly in bacterioplankton communities [81,82]. Although sometimes found in association with animal hosts [80], members of the family Saccharospiraceae have frequently been described as copiotrophs that participate the marine microbial loop as degraders in the catabolism of DSMP [83] or degradation of organic hydrocarbons, hence its close association with algal blooms [84].

The *Pantoea* genus consists of widely distributed bacterial species, found across diverse environments and forming associations with a wide range of hosts [85,86,87]. Previous studies have demonstrated that *Pantoea agglomerans* strain RCS2, isolated from healthy cobia fish (*Rachycentron canadum*), exhibited in vitro antagonistic activity against fish pathogens and possessed probiotic potential [88]. This species has been associated with significant enhancements in fish growth, feed efficiency, hematological parameters, survival under challenging conditions, and intestinal morphology [89,90]. Here, the abundance of *Pantoea* was mainly driven by ASV-4235, which was most similar (99.77%) to *Pantoea brenneri* (Table S4 in Supplementary File S3). Members of this species are commonly isolated from soil samples and have been shown to possess high fungicidal activity against plant pathogens and produce several metabolites with plant growth-promoting activities [91,92].

## 4. Conclusions

Currently there is a consensus that bacterial diversity in fish larvae generally increases with age until it peaks near the juvenile stage, irrespective of host species, rearing system, or feed regime [5]. However, our results suggest that fish size, in addition to age, can significantly influence the phylogenetic diversity of fish microbial communities. A more in-depth compositional analysis of dominant bacterial ASVs detected taxon-specific associations with particular fish categories. For example, *Aliivibrio* and *Sulfitobacter* were significantly enriched in smaller post-larvae, whereas Micrococcaceae were enriched in larger post-larvae. The genus *Exiguobacterium* was slightly more enriched in depigmented post-larvae, while *Pantoea* was more prevalent in normal-sized post-larvae. Our findings highlight the need for further efforts to culture and study representative members of these taxa, with the goal of elucidating their specific roles in shaping post-larvae phenotypic traits. This knowledge can contribute to the development of innovative microbiome manipulation strategies that focus on manipulating members of these taxa to enhance fish health, reduce larval mortality, and improve the overall quality of fish produced in larviculture systems.

## Data Availability

Sequences used in this study were uploaded to the NCBI ShortRead Archive (BioProject PRJNA1097451; Biosamples SAMN40759064-SAMN40759163).

## Supplementary Materials

The following supporting information can be downloaded at: https://www.mdpi.com/article/10.3390/microorganisms12102014/s1, Supplementary File S1—Sample information file. Supplementary File S2—ASV abundance table. Supplementary File S3—File with Supplementary Tables S1–S4 and Supplementary Figure S1. Supplementary Table S1—Results from Kruskal-Wallis rank sum tests applied to diversity indicators Shannon H’ (Shannon), Peilou’s J (evenness), Fisher α (Fisher) and Faith’s phylogenetic diversity index (Faith). Four tests were conducted: one referent to the categories (Sml, Lg, DeP, Def and Nor) and the remaining referent to each traits categorized: size (large vs. small vs. normal size), skeletal deformation (deformed vs. not deformed) and pigmentation (malpigmented vs pigmented). Supplementary Table S2—Post-hoc analysis using Dunn’s nonparametric test of multiple comparisons of Faith’s phylogenetic diversity index following a significant Kruskal-Wallis test. *p*-value was adjusted using the Benjamini-Hochberg procedure (false discovery rate). Bold indicates significant results (*p* < 0.05). Supplementary Table S3—Results from the one-way anova applied to general linearized models of relative abundance of taxonomic groups with tweedie distribution. Only relevant taxonomic groups (mean relative abundance in either category > 1%) were tested. Only significant results are presented here. F—F-value, R2—residual, *p*—*p*-value, Sign—significance (*p* < 0.05—*, *p* < 0.01—**, *p* < 0.001—***). Supplementary Table S4—List of 50 most abundant ASV in the dataset and their taxonomic information. Sequence similarity search was done using BLAST algorithm against 16S sequences from NCBI curated RefSeq records. ASV are presented by their unique MD5 hashes (ASV ID), arbitrary numbering (#) and most resolute taxonomic classification using the SILVA classifier (version 138, released December 2019). Sequence similarity search results present description of closest relative and their respective accession number, similarity (Sim), source (if available) and reference (if available). n.a.—not available. Supplementary Figure S1—Photographs of sampled size representative of each category. Malformed: normal size fish with normal pigmentation and at least one detectable skeletal malformation; Small: small size fish with normal pigmentation with no skeletal malformation; Depigmented: normal size fish with irregular pigmentation and no skeletal malformation; Large: large size fish with normal pigmentation and no skeletal malformation; and Normal: normal size fish with normal pigmentation and no skeletal malformation. Supplementary File S4—Results of pairwise comparisons performed by calculating the estimated marginal means of glm models, which related these effects to the specific categories and their combina-tions using pairs() function from the emmeans package with *p*-value adjustment set to the false discovery rate (i.e., p.adjust = “fdr”). Only comparison with the Normal category were considered.
