# Supplementary material for: Association between Turbot (Scophthalmus maximus) Fish Phenotype and the Post-Larval Bacteriome
Source: Microorganisms. 2024 Oct 4;12(10):2014. doi: 10.3390/microorganisms12102014 (PMC11510086; doi:10.3390/microorganisms12102014)
Supplement: Supplementary file 1 [file microorganisms-12-02014-s001.zip › microorganisms-3160989-supplementary/Supplementary File S3_REV.pdf]

Table S1 – Results from Kruskal-Wallis rank sum tests applied to diversity indicators Shannon H' (Shannon), Peilou's J (evenness), Fisher α (Fisher) and Faith's phylogenetic diversity index (Faith). Four tests were conducted: one referent to the categories (Sml, Lg, DeP, Def and Nor) and the remaining referent to each traits categorized: size (large vs. small vs. normal size), skeletal deformation (deformed vs. not deformed) and pigmentation (malpigmented vs pigmented)

Test	Diversity Index	$H_{1,4}$	P-value
Categories	Shannon	0.907	0.923
	Evenness	0.782	0.941
	Fisher	6.272	0.18
	**Faith**	**12.309**	**0.015**

Table S2 – Post-hoc analysis using Dunn's nonparametric test of multiple comparisons of Faith's phylogenetic diversity index following a significant Kruskal-Wallis test. P-value was adjusted using the Benjamini-Hochberg procedure (false discovery rate). Bold indicates significant results (P < 0.05).

Comparison	Z-value	P-value	Adjusted P-value
Large – Malformed	-0.867	0.389	0.778
Large – Normal	-0.758	0.449	0.641
Malformed – Normal	0.104	0.918	1.00
**Large – Small**	**-3.047**	**0.002**	**0.023**
**Malformed – Small**	**-2.185**	**0.029**	0.072
**Normal – Small**	**-2.289**	**0.022**	0.074
Large – Depigmented	-0.049	0.961	0.961
Malformed – Depigmented	0.812	0.417	0.695
Normal – Depigmented	0.709	0.479	0.598
**Small – Depigmented**	**2.998**	**0.003**	**0.014**

*Table S3 -Results from the one-way anova applied to general linearized models of relative abundance of taxonomic groups with tweedie distribution. Only relevant taxonomic groups (mean relative abundance in either category > 1%) were tested. Only significant results are presented here. F- F-value, R2 – residual, P – p-value, Sign – significance (P < 0.05 - *, P < 0.01 - **, P < 0.001 - ***).*

Taxa	$F_{4,95}$	Resid. Dev.	P	Sign	Taxa	$F_{4,95}$	Resid. Dev.	P	Sign
Bacteroidota	3.646	3552.1	0.006	**	Flavobacteriaceae	4.239	2960.8	0.002	**
Alphaproteobacteria	3.706	7426.6	0.005	**	Micrococcaceae	4.979	13214	<0.001	***
Bateroidia	3.64	3552	0.006	**	Planococcaceae	6.067	4066	<0.001	***
Alteromonadales	3.231	2784.7	0.012	*	Rhodobacteraceae	4.455	7249	0.001	**
Cytophagales	10.272	3495.2	<0.001	***	Saccharospirillaceae	4.548	4621.6	0.001	**
Enterobacterales	9.565	4993.7	<0.001	***	*Aliivibrio*	4.23	7975.6	0.002	**
Exiguobacterales	4.158	5088.7	0.002	**	*Domibacillus*	9.975	2977.1	<0.001	***
Flavobacteriales	3.892	2901.6	0.004	**	*Exiguobacterium*	4.1584	5088.7	0.002	**
Micrococcales	4.327	13275	0.002	**	NS10_marine_group	2.705	3911.1	0.029	*
Oceanospirillales	3.601	3754.4	0.006	**	*Pantoea*	11.202	4770.9	<0.001	***
Rhodobacterales	4.4554	7249.0	0.001	**	*Polaribacter*	2.555	3710.4	0.037	*
Colwelliaceae	2.938	3784.5	0.02	*	*Sulfitobacter*	4.799	11540	<0.001	***
Erwiniaceae	11.243	4796.9	<0.001	***	*Aliivibrio fischeri*	4.2651	8052.9	0.002	**
Exiguobacteraceae	4.1584	5088.7	0.002	**	*Vibrio furnissii*	3.508	4771.4	0.007	**

Table S4 – List of 50 most abundant ASV in the dataset and their taxonomic information. Sequence similarity search was done using BLAST algorithm against 16S sequences from NCBI curated RefSeq records. ASV are presented by their unique MD5 hashes (ASV ID), arbitrary numbering (#) and most resolute taxonomic classification using the SILVA classifier (version 138, released December 2019). Sequence similarity search results present description of closest relative and their respective accession number, similarity (Sim), source (if available) and reference (if available). n.a.-not available

			Sequence Similaity Search				
#	ASV ID	Silva	Closest relative	Acession number	Sim	Source	Reference
7	1a9301a03c70f53dc126bbf6b1aada9e	*Vibrio*	*Vibrio gigantis* strain LGP 13	NR_114910.1	100	Haemolyph of *Crassostrea gigas*	(Roux et al. 2005)
1	4a7f867d55034fd3b2b4b28e0b1dda36	*Rhodococcus*	*Rhodococcus qingshengii* strain djl-6-2	NR_115708.1	100	Wastewater sludge	(Wang et al. 2010)
25	d026ba8391312cd4726993268770b541	*Ralstonia*	*Ralstonia syzygii* subsp. *indonesiensis* strain UQRS	NR_134150.1	98.36	Tomato plant	n.a.
4	d72da071bc9d900b190163128cb16283	*Stenotrophomonas*	*Stenotrophomonas geniculata* ATCC 19374	NR_024708.1	99.53	Tap water	n.a.
13	e126d0d3db2858c647557595da6b24e9	*Vibrio*	*Vibrio gigantis* strain LGP 13	NR_114910.1	99.53	Haemolyph of *Crassostrea gigas*	(Roux et al. 2005)
14	0ca1de0b0d71831974a9aa9516f27dfc	*Stenotrophomonas*	*Stenotrophomonas geniculata* ATCC 19374	NR_024708.1	100	Tap water	n.a.
57	c115905e9fcddb82a523014b244586e9	*Sulfitobacter*	*Sulfitobacter*	NR_165714	97.76	Deep seawater	(Song et al.

			profundi strain SAORIC-263				2019)
12	6d66f24ce1ac56865b36295b131385f5	*Vibrio*	*Vibrio cyclitrophicus* strain LMG 21359	NR_115806	100	creosote-contaminated marine sediment	(Hedlund and Staley 2001)
38	d1316b26e5350da36e6456499ecdfe18	Micrococcaceae	*Pseudarthrobacter phenanthrenivorans Sphe3*	NR_074770	100	creosote polluted site	(Kallimanis et al. 2011)
5	4a5ef3b18f362cc8c3d5ea749ca7831e	*Vibrio*	*Vibrio lentus* strain CIP 107166	NR_114982	100	oyster	(Dias et al. 2018)
4639	2fd1a56439ee54b64c57aa5433e18d9d	*Vibrio*	*Vibrio fluvialis* strain NCTC 11327	NR_118443	100	Human faeces	
59	a2ca82df92ebb0a8a398a24239dcc26c	*Vibrio*	*Vibrio scophthalmi* LMG 19158	NR_117889	100	isolated from a marine sponge (Scleritoderma cyanea)	(Hoffmann et al. 2012)
53	e012d8ef9cdb69a1b6c7bd303ac3b136	*Vibrio*	*Vibrio sinensis* strain BEI233	NR_179929	99.06	Surface seawater of the East China Sea	n.a.
8	13008322aeaf6d05249ad9682125b366	*Vibrio*	*Vibrio parahaemolyticus* strain ATCC 17802	NR_118569	100	food poisened victim	n.a.
8867	0e580049de0eb9d459473f891c191c30	*Aliivibrio*	*Aliivibrio fischeri* ATCC 7744	NR_115204	97.42	seawater	n.a.
44	8b4e1dd8ce89f7406fe32819e1f336b6	*Vibrio*	*Vibrio hyugaensis*	NR_145569	100	seawater	n.a.

			strain 090810ª				
10	58a4607c04de196174b57b1080377325	*Polaribacter*	*Polaribacter marinaquae* strain RZW3-2	NR_152672	100	Seawater	(Wang et al. 2016)
1820	946f1b3bf75d231e55cd9294801a6c41	*Vibrio*	*Vibrio kanaloae* strain LMG 20539	NR_042468	100	diseased oyster larvae	(Dias et al. 2018)
4235	f87045bf18de1e8f760333c4b6e37c03	*Pantoea*	*Pantoea brenneri* strain LMG 5343	NR_116748	99.77	human urethra	(Brady et al. 2010)
4250	5727d3487e755c7535a608fe7c7c2627	*Exiguobacterium*	*Exiguobacterium qingdaonense* strain S82	NR_181609	100	Sargussum	(Liu et al. 2021)
2751	b058fbb3253342d65d0f844fb22d205a	*Vibrio*	*Vibrio fluvialis* strain NCTC 11327	NR_118443.1	99.77	human faeces, diarrhoea	n.a.
11	9b796e9407c2fad1a2326d05e8c202e4	*Rhodobacteraceae*	*Phaeobacter porticola* strain P97	NR_157650.1	99.5	Seawater, port environment	(Breider et al. 2017)
5188	9fc0bc3dd97a1285c0ca73b11048afa5	*Vibrio*	*Vibrio furnissii* strain 9119-82	NR_037067	100	Human feces	(Brenner et al. 1983)
17	f068d1b23ce6707fc7b98827e7c03943	*Flavobacteriaceae*	*Polaribacter aestuariivivens* strain DBTF-3	NR_180191.1	97.39	Tidal flat	(Park et al. 2019)
34	15da8b3417459a1a66342300e6968701	*Methylobacterium-Methylorubrum*	*Methylobacterium bullatum* strain F3.2	NR_117561	100	Phyllosphere of moss *Funaria hygrometrica*	(Hoppe et al. 2011)
68	be8ce2ecf5b77c82dad4c841aead5285	*Saccharospirillaceae*	*Spongiispira norvegica* strain	NR_042453.1	97.66	boreal sponge *Isops phlegraei*	(Kaesler et al. 2008)

			Gp_4_7.1				
8092	d40450630dcb89c3cb4e005547673556	*Vibrio*	*Vibrio scophthalmi* LMG 19158	NR_117889.1	99.77	isolated from a marine sponge (Scleritoderma cyanea)	(Hoffmann et al. 2012)
6194	2602adef86f7951782017a49ae7e6ea1	*Vibrio*	*Vibrio furnissii* strain 9119-82	NR_037067	99.53	Human feces	(Brenner et al. 1983)
769	0a1f5ed70dcb37a86924dbd5fde981af	*Enhydrobacter*	*Moraxella osloensis* strain A1920	NR_104936.1	100	Human cerebrospinal fluid	n.a.
5493	441101e5a1903427304d82ca281c930f	*Vibrio*	*Vibrio chagasii* strain LMG 21353	NR_117891.1	100	isolated from a marine sponge (Scleritoderma cyanea)	(Hoffmann et al. 2012)
63	47f0dfa872ad06bbe11ca59b31acc51c	*Vibrio*	*Vibrio alginolyticus* strain NBRC 15630	NR_122050.1	99.53	diseased cobia Rachycentron canadum	(Liu et al. 2004)
6	25865e882d21e0aa6fffa300375fc3ac	*Thalassobius*	*Thalassovita mediterranea* strain XSM19	NR_042377.1	99.25	Seawater	(Arahal et al. 2005)
2075	28bedfee1c832d07948a7b261e73e724	*Domibacillus*	*Domibacillus indicus* strain SD111	NR_134022.1	100	Marine sediments	(Sharma et al. 2014)
94	9a14381fb9d27cd274ef3d057292876e	*Thalassotalea*	*Thalassotalea loyana* strain CBMAI 722	NR_043066	7.19	Diseased corals	(Thompson et al. 2006)

2440	226bd8e858ee0396ea6909d499e96866	*Aliivibrio*	*Aliivibrio fischeri* strain 398	NR_029255	100		
1778	5497318e515a8c328a68f95975d9c7d4	*Staphylococcus*	*Staphylococcus caprae* strain DSM 20608	NR_119252.1	100	blood cultures of a neonate	(Spellerberg et al. 1998)
4725	1a583fc2d950f287db623739e6b63511	*Vibrio*	*Vibrio furnissii* strain 9119-82	NR_037067	99.77	Human feces	(Brenner et al. 1983)
79	e688e029724fdfdd9cd096df2c443740	*Amylibacter*	*Amylibacter ulvae* strain KMM 6515	NR_146351	100	Pacific green alga Ulva fenestrata	(Nedashkovskaya et al. 2016)
43	136e3c7e080e39beda14e9bc9bb7b59f	*Vibrio*	*Vibrio splendidus* strain AlyHP32	NR_109668	99.53	Sea urchin (Hemicentrotus pulcherrimus)	(Kim et al. 2013)
5253	d83a14ea274cb3e35b91f494c87d4c90	*Burkholderia-Caballeronia-Paraburkholderia*	*Paraburkholderia podalyriae* strain WC7.3b	NR_178424.1	99.3	Papilionoid legumes nodules	(Beukes et al. 2013)
6065	5db606dba3a142c4b04e1ee4c4e36c74	*Polaribacter*	*Polaribacter marinaquae* strain RZW3-2	NR_152672	99.76	Seawater	(Wang et al. 2016)
6686	22ffdb82a39c73a8402594095c3e4c88	*Kineosporiaceae*	*Kineococcus xinjiangensis* strain S2-20	NR_044522	98.53	Desert sand	(Liu et al. 2009)
1984	f1f17c067df6e4c151cd865400923d27	*Pseudoalteromonas*	*Pseudoalteromonas marina* strain mano4	NR_042981.1	100	Tidal flats	(Nam et al. 2007)
5800	866e65b9a77759e6f7d7fff8ec2bc416	*Vibrio*	*Vibrio kanaloae* strain LMG 20539	NR_042468.1	99.77	Clam Dosinia exoleta	(Dias et al. 2018)

6176	3362c840d7ca5a9793f2dc71668ef025	*Bernardetia*	*Bernardetia litoralis* DSM 6794	NR_102950	97.16	Seawater	(Lewin and Lounsbery 1969)
2591	62611dedc42201591d0c89973f903dde	*Staphylococcus*	*Staphylococcus hominis* subsp. *novobiosepticus* strain GTC 1228	NR_041323.1	100	n.a.	n.a.
39	5072827e092a66498374bee9b9c7d0fa	*Vibrio*	*Vibrio ichthyoenteri* ATCC 700023	NR_117888.1	99.3	Gut of diseased Japanese flounder larvae	(Hoffmann et al. 2012)
3871	0aed2064b15be56ad488bdd263d4e1ce	*Pseudophaeobacter*	*Polaribacter marinaquae* strain RZW3-2	NR_152672	99.76	Seawater	(Wang et al. 2016)
7980	5a5b232c41f47a6a0cc826af358b0b66	*Vibrio*	*Vibrio fluvialis* strain NCTC 11327	NR_118443.1	99.53	Human faeces, diarrhoea	n.a..
7	c94875dc025f74bd2fe2df6f8f6c4abe	*Vibrio*	*Vibrio gigantis* strain LGP 13	NR_114910.1	100	Haemolyph of *Crassostrea gigas*	(Roux et al. 2005)

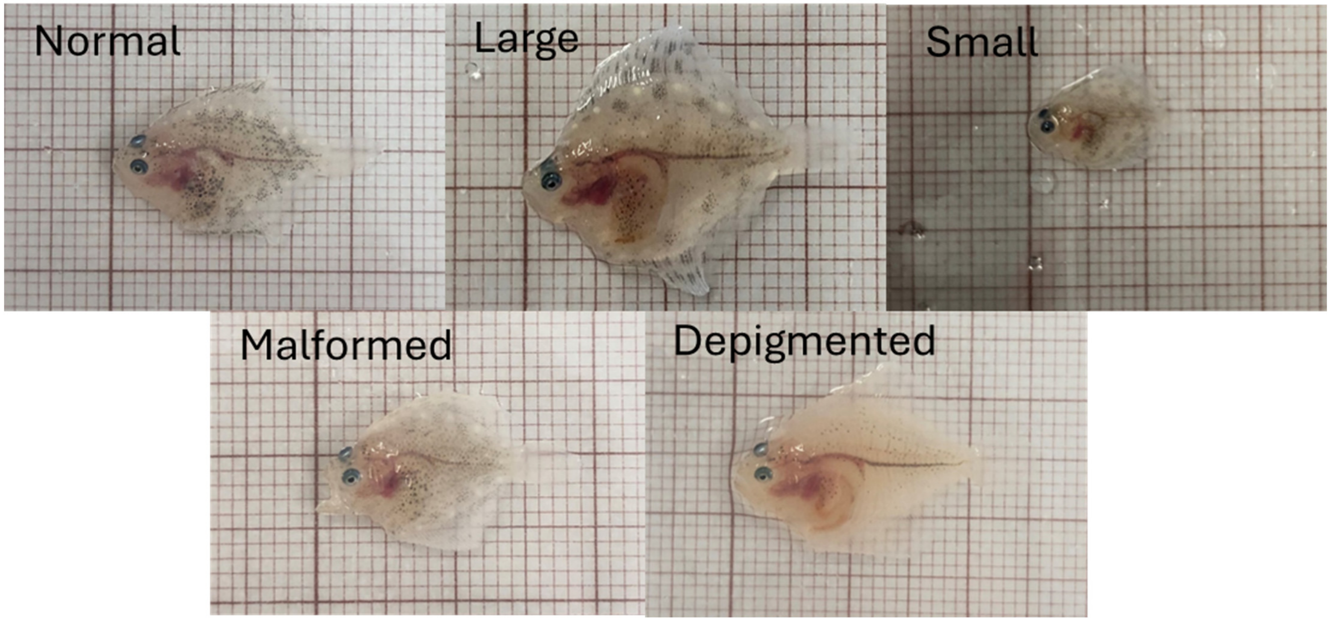

Supplementary Figure S1 – Photographs of sampled size representative of each category. Malformed: normal size fish with normal pigmentation and at least one detectable skeletal malformation; Small: small size fish with normal pigmentation with no skeletal malformation; Depigmented: normal size fish with irregular pigmentation and no skeletal malformation; Large: large size fish with normal pigmentation and no skeletal malformation; and Normal: normal size fish with normal pigmentation and no skeletal malformation.

Arahal DR, Macián MC, Garay E, Pujalte MJ (2005) Thalassobius mediterraneus gen. nov., sp. nov., and reclassification of Ruegeria gelatinovorans as Thalassobius gelatinovorus comb. nov. Int J Syst Evol Microbiol 55:2371–2376

Beukes CW, Venter SN, Law IJ, et al (2013) South African papilionoid legumes are nodulated by diverse Burkholderia with unique nodulation and nitrogen-fixation loci. PLoS One 8:e68406

Brady CL, Cleenwerck I, Venter SN, et al (2010) Emended description of the genus Pantoea, description of four species from human clinical samples, Pantoea septica sp. nov., Pantoea eucrina sp. nov., Pantoea brenneri sp. nov. and Pantoea conspicua sp. nov., and transfer of Pectobacterium cypripedii (Hori 1911) Brenner et al. 1973 emend. Hauben et al. 1998 to the genus as Pantoea cypripedii comb. nov. Int J Syst Evol Microbiol 60:2430–2440. https://doi.org/10.1099/ijs.0.017301-0

Breider S, Freese HM, Spröer C, et al (2017) Phaeobacter porticola sp. nov., an antibiotic-producing bacterium isolated from a sea harbour. Int J Syst Evol Microbiol 67:2153–2159. https://doi.org/10.1099/ijsem.0.001879

Brenner DJ, Hickman-Brenner FW, Lee J V, et al (1983) Vibrio furnissii (formerly aerogenic biogroup of Vibrio fluvialis), a new species isolated from human feces and the environment. J Clin Microbiol 18:816–824. https://doi.org/10.1128/jcm.18.4.816-824.1983

Dias GM, Bidault A, Le Chevalier P, et al (2018) Vibrio tapetis displays an original type IV secretion system in strains pathogenic for Bivalve molluscs. Front Microbiol 9:227

Hedlund BP, Staley JT (2001) Vibrio cyclotrophicus sp. nov., a polycyclic aromatic hydrocarbon (PAH)-degrading marine bacterium. Int J Syst Evol Microbiol 51:61–66

Hoffmann M, Monday SR, Allard MW, et al (2012) Vibrio caribbeanicus sp. nov., isolated from the marine sponge Scleritoderma cyanea. Int J Syst Evol Microbiol 62:1736–1743. https://doi.org/10.1099/ijs.0.032375-0

Hoppe T, Peters K, Schmidt F (2011) Methylobacterium bullatum sp. nov., a methylotrophic bacterium isolated from Funaria hygrometrica. Syst Appl Microbiol 34:482–486. https://doi.org/https://doi.org/10.1016/j.syapm.2010.12.005

Kaesler I, Graeber I, Borchert MS, et al (2008) Spongiispira norvegica gen. nov., sp. nov., a marine bacterium isolated from the boreal sponge Isops phlegraei. Int J Syst Evol Microbiol 58:1815–1820

Kallimanis A, LaButti KM, Lapidus A, et al (2011) Complete genome sequence of Arthrobacter phenanthrenivorans type strain (Sphe3). Stand Genomic Sci 4:123–130. https://doi.org/10.4056/sigs.1393494

Kim D, Baik KS, Hwang YS, et al (2013) Vibrio hemicentroti sp. nov., an alginate lyase-producing bacterium, isolated from the gut microflora of sea urchin (Hemicentrotus pulcherrimus). Int J Syst Evol Microbiol 63:3697–3703

Lewin RA, Lounsbery DM (1969) Isolation, Cultivation and Characterization of Flexibacteria. J Gen Microbiol 58:145–170. https://doi.org/10.1099/00221287-58-2-145

Liu F, Li Y, He W, et al (2021) Exiguobacterium algae sp. nov. and Exiguobacterium qingdaonense sp. nov., two novel moderately halotolerant bacteria isolated from the coastal algae. Antonie Van Leeuwenhoek 114:1399–1406. https://doi.org/10.1007/s10482-021-01594-8

Liu M, Peng F, Wang Y, et al (2009) Kineococcus xinjiangensis sp. nov., isolated from desert sand. Int J Syst Evol Microbiol 59:1090–1093

Liu P-C, Lin J-Y, Hsiao P-T, Lee K-K (2004) Isolation and characterization of pathogenic Vibrio alginolyticus from diseased cobia Rachycentron canadum. J Basic Microbiol 44:23–28. https://doi.org/https://doi.org/10.1002/jobm.200310316

Nam Y-D, Chang H-W, Park JR, et al (2007) Pseudoalteromonas marina sp. nov., a marine bacterium isolated from tidal flats of the Yellow Sea, and reclassification of Pseudoalteromonas sagamiensis as Algicola sagamiensis comb. nov. Int J Syst Evol Microbiol 57:12–18

Nedashkovskaya OI, Kukhlevskiy AD, Zhukova N V, Kim SB (2016) Amylibacter ulvae sp. nov., a new alphaproteobacterium isolated from the Pacific green alga Ulva fenestrata. Arch Microbiol 198:251–256

Park S, Park J-M, Lee J-S, Yoon J-H (2019) Description of Polaribacter aestuariivivens sp. nov., isolated from a tidal flat. FEMS Microbiol Lett 366:fnz185. https://doi.org/10.1093/femsle/fnz185

Roux F Le, Goubet A, Thompson FL, et al (2005) Vibrio gigantis sp. nov., isolated from the haemolymph of cultured oysters (Crassostrea gigas). Int J Syst Evol Microbiol 55:2251–2255

Sharma A, Dhar SK, Prakash O, et al (2014) Description of Domibacillus indicus sp. nov., isolated from ocean sediments and emended description of the genus Domibacillus. Int J Syst Evol Microbiol 64:3010–3015

Song J, Jang H-J, Joung Y, et al (2019) Sulfitobacter profundi sp. nov., isolated from deep seawater. Journal of Microbiology 57:661–667

Spellerberg B, Steidel K, Lütticken R, Haase G (1998) Isolation of Staphylococcus caprae from blood cultures of a neonate with congenital heart disease. European Journal of Clinical Microbiology and Infectious Diseases 17:61–62

Thompson FL, Barash Y, Sawabe T, et al (2006) Thalassomonas loyana sp. nov., a causative agent of the white plague-like disease of corals on the Eilat coral reef. Int J Syst Evol Microbiol 56:365–368

Wang Y, Gao L, Ming H, et al (2016) Polaribacter marinaquae sp. nov., isolated from seawater. Int J Syst Evol Microbiol 66:4594–4599

Wang Z, Xu J, Li Y, et al (2010) Rhodococcus jialingiae sp. nov., an actinobacterium isolated from sludge of a carbendazim wastewater treatment facility. Int J Syst Evol Microbiol 60:378–381
